# Evaluation of Patient Satisfaction and Clinical Efficacy of Using Blood Glucose Meters Featuring Color-Coded Indicators in Patients With Type 1 Diabetes: An Observational Hospital-Based Study

**DOI:** 10.7759/cureus.23764

**Published:** 2022-04-02

**Authors:** Ayman Al Hayek, Mohamed Al Dawish

**Affiliations:** 1 Endocrinology and Diabetes, Prince Sultan Military Medical City, Riyadh, SAU

**Keywords:** glucose monitoring, saudi arabia., medisafe fit smile, color-coded range indicators, type 1 diabetes

## Abstract

Introduction

Because of the difficulties in interpreting the level of blood glucose using the traditional numeric glucometers, the blood glucose meters featuring color-coded range indicators (CRI) offered a simple way to understand and interpret blood glucose readings. Therefore, this study aimed to assess glucose monitoring satisfaction (GMS) and clinical efficacy of blood glucose meters featuring color-coded in patients with Type 1 Diabetes (T1DM) in Saudi Arabia.

Methods

T1DM patients who switched to color-coded Medisafe Fit Smile glucometer were included in this study. Demographic data, clinical characteristics, glycemic parameters were collected. A trained interviewer collected the GMS survey at baseline and 12 weeks. At the end of the study, patient-reported satisfaction with the Medisafe Fit Smile color-coded features (MSCF) survey was collected.

Results

This study included 62 T1DM patients with a mean age of 17.8 (±3.1), and the majority had three or more confirmed hypoglycemic episodes per month. Compared to baseline data, we showed a significant improvement in the mean hemoglobin A1C level (8.43 [±1.2] versus 8.36 [±1.1], p<0.001), with a significantly lower frequency of hypoglycemic episodes (2.74 [±1.1] versus 2.0 [±0.78], p<0.001) after 12 weeks of using glucometer featuring CRI. Most importantly, significant improvements in the GMS survey subdomains of openness (p<0.001), emotional burden (p<0.001), behavioral burden (p<0.001), but not trust (p=0.71) were detected at the end of the study with a significantly higher total GMS survey of 4.33 (±0.13) after using blood glucose meters featuring CRI compared to the lower score of 2.84 (±0.22) at baseline (p<0.001). Furthermore, patient satisfaction with the MSCF survey revealed evidence of satisfaction among the TIDM patients at the end of the study.

Conclusion

The current study confirmed that individuals with T1DM may benefit from blood glucose meters featuring CRI device exposure. Also, using a glucometer featuring CRI was associated with a high level of satisfaction with blood glucose measures and significant improvement in the glycemic parameters. However, definitely, further studies are needed to confirm whether the long-term use of the CRI-based blood glucose meters will produce improved results in the GMS survey.

## Introduction

Improving the quality of care for patients with diabetes mellitus (DM) has become a major priority for the health care system and policymakers [1]. Also, patient satisfaction is becoming highly significant and largely acknowledged as a key indicator of the quality of the healthcare system [2]. Because hypoglycemia can be associated with an increased risk of mortality and a negative impact on patients' quality of life and satisfaction with diabetes management, self-monitoring of blood glucose (SMBG) is important for glycemic control and also help physicians to accurately develop dietary and diabetes drug therapy recommendations [3]. The importance of SMBG in the management of type 1 diabetes mellitus (T1DM) patients is undeniable, and the link between increased SMBG frequency and decreased hemoglobin A1C levels (HbA1C) has been established, especially with those on insulin therapy [4,5].

On the other hand, various limitations to the effective use of SMBG exist, including but not limited to a lack of patient understanding of when to test their blood glucose, how to interpret glucose readings, and how to deal with any abnormal values [6,7]. Shortly following a diabetes diagnosis, patients are routinely educated on how to read blood glucose levels. However, it is obvious that glucose range awareness must be reinforced on a regular basis, particularly the response to "out of range" levels of blood glucose [8].

In Saudi Arabia, recent research has brought attention to the problem of interpreting and responding to SMBG. A cross-sectional study in 207 patients with T1DM investigating factors and barriers that affect patients' compliance to SMBG demonstrated that lack of availability of information to deal with blood glucose reading is one of the influential factors affecting compliance to SMBG and is associated with more diabetes-related complications [9]. Moreover, a study of 866 diabetic patients revealed that about 54% of insulin users and 56% of non-insulin users did not take action for either low or high blood glucose levels [10].

Modern technologies for diabetic care management have been implemented to ease the burden of SMGB to provide better glycemic control and satisfaction for T1DM patients. In that respect, the blood glucose meters featuring color-coded range indicators (CRI) as Medisafe Fit Smile [11] offered a simple way to understand and interpret blood glucose readings by displaying colored lights that indicate whether the reading is above, within, or below the intended range. Grady et al.'s study showed that the use of blood glucose meters featuring CRI can improve glycemic control in patients with diabetes [12].

Furthermore, our recently published study showed the use of blood glucose meters featuring CRI was associated with improving the patients’ satisfaction with blood glucose measures in T2DM patients [13]. In the present study, we aimed to assess the patient satisfaction and clinical efficacy of blood glucose meters featuring color-coded in patients with T1DM in Saudi Arabia.

## Materials and methods

Study design and procedures

We conducted a single-center prospective observational study at the department of endocrinology and diabetes, diabetes treatment center, Prince Sultan Military Medical City (PSMMC), Riyadh.

We included a convenience sample of 62 diabetic patients who attended our center from March to July 2021 in this study. The study protocol was approved by the Research and Ethics Committee of PSMMC (IRB approval No.# 1310). After clearly explaining the objectives and research methodology, all participants provided oral and written informed consent before completing the study measurement. For patients ≤18 years of age, verbal consent from the patients and written informed consent from their parents/caregivers were obtained.

Study population

We considered any T1DM patient attending our department at PSMMC who fulfilled the following inclusion criteria: (1) T1DM patients aged 14-40 years treated with multiple insulin doses using basal-bolus therapy for at least one year, (2) patients who used their usual conventional SMBG to self-test their glucose levels at least two times a day for at least six months before enrollment, and (3) agreed to participate in the study and sign the informed consent form. We excluded patients who were registered for another clinical study.

Study procedures

At the baseline visit, eligible patients with T1DM have signed the informed consent and switched to using a blood glucose meter (BGM) featuring CRI (Medisafe Fit Smile, Terumo Corporation).

The following data were collected and documented in a standardized case record form: (1) demographic information and clinical (age, gender, weight, and BMI) and clinical characteristics (DM duration, BGM frequency, total daily insulin dose (TDD), confirmed hypoglycemia episode in the past three months, and HbA1c). All patients have received a Medisafe Fit Smile glucometer at baseline visit to self-monitor their blood glucose levels.

At the time of the baseline visit and 12 weeks of the study, a trained interviewer introduced Glucose Monitoring Satisfaction Survey (GMSS) [14] to every patient to test glucose monitoring satisfaction. In addition, patient-reported satisfaction with the Medisafe Fit Smile color-coded features (MSCF) survey was collected at the final site visit [15]. 

Study outcomes

The primary outcome of this study was to assess the difference in the patient's glucose monitoring satisfaction before and after switching to a glucometer featuring CRI device (baseline versus 12-week) using GMSS scores. In addition, MSCF survey was used at the end of the study to assess the participant's Medisafe Fit Smile perception at the end of the study (after 12 weeks). The secondary outcome was to address the impact of using BGMs featuring CRI on the HbA1c level, frequency of hypoglycemic episodes, and TDD (baseline versus 12-week).

Assessment of the patient satisfaction

The GMSS subdomains (openness, emotional burden, behavioral burden, and trust) were used to assess the level of glucose monitoring satisfaction through the T1DM version of the survey (GMSS version T1DM) [14]. Each subdomain can be obtained by calculating the mean item response score for the following subdomains. The openness subdomain represents questions number 1, 8, 10, and 14, the emotional burden subdomain represents questions number 2, 5, 9, and 13, the behavioral burden subdomain represents questions number 3, 6, 11, and 15, trust subdomain represents the questions number 4, 7, and 12 (Reverse code items were used in the trust subdomain). A five-point Likert-type scale ranging from one (strongly disagree) to five (strongly agree) was used for rating the response to each item and was employed where higher scores showed higher GMS.

Alternatively, the MSCF survey includes 12 questions to measure the participant's Medisafe Fit Smile perception. A trained interviewer asked the patients to rate their experience with the system on a scale ranging from one (strongly agree) to five (strongly disagree) [15].

Assessment of HbA1c and hypoglycemia

HbA1c levels were tested at baseline visit and after 12 weeks (end of study) using the COBAS NTEGRA® 400 plus/800 analyzer (Roche Diagnostics, USA) at PSMMC central laboratory. A confirmed blood glucose level of ≤ 70 mg/dL was considered hypoglycemia.

Statistical analysis

The data were analyzed using SPSS software (version 25). All categorical variables were presented in frequency and percentage, whereas the continuous variables were presented with descriptive statistics (median, mean, SD, and range). To compare the baseline data and the outcome variables on a continuous scale, paired t-test was used for paired and normally distributed data comparisons to determine the differences between the different time points (baseline versus 12 weeks). However, the chi-square test was used for categorical data comparisons. An alpha level below 0.05 was used to indicate statistical significance.

The sample size for this study was calculated using Statulator software [16]. Based on identifying a variance of one point in the glucose monitoring satisfaction total test scores between baseline and 12 weeks (paired comparison) with 95% confidence and 80% power and consider a standard deviation of 2.5 in differences between baseline and 12 weeks, a minimum of 52 patients were required for this study with a level of significance of 5% (two-sided). Considering a drop-out rate of 10% total sample size required is 60 patients

## Results

This study included 62 T1DM patients with a mean age of 17.8 (±3.1), and 54.8% of the study sample were female. The demographic characteristics of the study population are shown in ​Table1. The majority of the study population (69.4%) was diagnosed with diabetes for <10 years with a mean BMI of 24.8 (±3.03) and HbA1c of 8.43 (±1.2). In addition, most of the included patients (64.8%) had three or more confirmed hypoglycemic episodes per month, with a TDD of 1.02 (±0.24) U/Kg/day. 

**Table 1:** Baseline demographic and clinical characteristics of the study population. Note: SD = standard deviation, BMI = body mass index, BGM = blood glucose meter, TDD = total daily insulin dose.

Variables	Values	
Age, year	Mean (±SD), Range	17.8 (±3.1), [14 – 29]
	< 20 years	51 (82.3%)
	≥20 years	11 (17.7%)
Gender, n (%)	Male	28 (45.2%)
	Female	34 (54.8%)
Weight, kg	Mean (±SD), Range	61.1 (±7.8), [40 – 80]
BMI, kg/m^2^	Mean (±SD), Range	24.8 (±3.03), [19.1 – 32.4]
	< 25 kg/m^2^	39 (62.9%)
	25 to < 30 kg/m^2^	16 (25.8%)
	≥ 30 kg/m^2^	7 (11.3%)
Diabetes duration, years	Mean (±SD), Range	8.3 (±3.1), [3 – 21]
	< 10 years	43 (69.4%)
	≥ 10 years	19 (30.6%)
BGM frequency, n (%)	2	49 (79%)
	3	13 (21%)
Confirmed hypoglycemia episode/month, n (%)	1	7 (11.3%)
	2	23 (37.1%)
	3	14 (22.6%)
	4	15 (24.2%)
	5	3 (4.8%)
TDD, unit/kg/Day	Mean (±SD), Range	1.02 (±0.24), [0.5 – 1.4]
	< 1 U/kg/day	29 (46.7%)
	≥ 1 U/kg/day	33 (53.3%)
HbA1c, %	Mean (±SD), Range	8.43 (±1.2), [6.9 – 13]

Table 2 shows the influence of using glucometer featuring CRI device on the glycemic parameters and the frequency of hypoglycemia. The mean HbA1c level has significantly improved after 12 weeks of using glucometer featuring CRI device compared to the baseline value. The mean frequency of confirmed hypoglycemia at baseline was 2.74 (±1.1), which decreased to 2.0 (±0.78) episodes after 12 weeks (p-value <0.001). About 51.6% of the included patients reported more than two episodes of hypoglycemia at baseline, which decreased significantly to only 20.9% (p<0.001).

**Table 2:** Influence of Medisafe Fit Smile Blood Glucose Meter on HbA1c, frequency of hypoglycemia, and SMBG frequency Note: TDD = total daily insulin dose, BGM = blood glucose meter.

	Baseline	After 12 weeks	P value
HbA1c, %	8.43 (±1.2)	8.36 (±1.1)	<0.001
TDD, unit/kg/Day	.02 (±0.24)	0.96 (±0.18)	<0.001
BGM frequency	1.94 (±0.69)	3.26 (±0.72	<0.001
BGM frequency > 2 times	13 (20.1%)	55 (88.7%)	<0.001
Confirmed hypoglycemia	2.74 (±1.1)	2 (±0.78)	<0.001
Confirmed hypoglycemia > 2 episodes	32 (51.6%)	13 (20.9%)	0.0008

**Figure 1:**
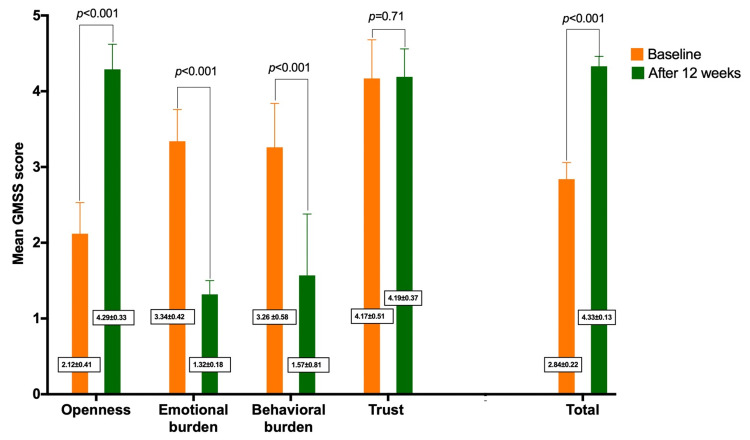
Patient satisfaction with Medisafe Fit Smile Blood Glucose Meter assessed by glucose monitoring satisfaction subdomains scores

Regarding the patient's glucose monitoring satisfaction at baseline versus after 12 weeks of switching to glucometer featuring CRI device using GMSS, the comparison revealed a significant improvement in the subdomains of openness (p<0.001), emotional burden (p<0.001), behavioral burden (p=0.0001), with the total GMSS score 2.84 (±0.22) at baseline versus 4.33 (±0.13) after 12 weeks, p<0.001. While the subdomains of trust showed no significant results (4.17±0.51, at baseline versus 4.19 ±0.37, after 12 weeks, p=0.71), as seen in Figure 1. Furthermore, patient satisfaction with MSCF survey at the end of the study showed that 60% of the patients strongly agreed that the use of green color for an in-range blood glucose result was intuitive and easy to understand, while 40.3% strongly agreed and 54.8% agreed that the use of red color for a high blood glucose result is intuitive and easy to understand. Also, 63% of the patients agreed that the Medisafe Fit Smile color-coded indicator was helpful for identifying low and high blood glucose results. About 50% of the patients strongly agreed, and 32% agreed to recommend the Medisafe Fit Smile meter, while 72.6% strongly agreed to switch to a meter with a color-coded indicator feature. The detailed result of the MSCF survey was reported in Table 3.

**Table 3:** Patient-reported satisfaction with Medisafe Fit Smile color-coded features survey statements after 12 weeks

No.	Survey statements	Strongly agree	Agree	Neither agree nor disagree	Disagree	Strongly disagree
1	The use of green for an in-range blood glucose result is intuitive and easy to understand	37 (59.7%)	21 (33.9%)	4 (6.5%)	0	0
2	The Medisafe Fit Smile™ color coded indicator is helpful for identifying low and high blood glucose result	19 (30.6%)	39 (62.9%)	4 (6.5%)	0	0
3	The use of Red for a high blood glucose result is intuitive and easy to understand	25 (40.3%)	34 (54.8%)	2 (3.2%)	1 (1.6%)	0
4	The Medisafe Fit Smile™ color coded indicator feature provides visual assurance of my blood glucose result	34 (54.8%)	27 (43.5%)	1 (1.6%)	0	0
5	I would recommend Medisafe Fit Smile™ meter	31 (50%)	20 (32.3%)	11 (17.7%)	0	0
6	I would switch to a meter with color coded indicator feature	45 (72.6%)	16 (25.8%)	1 (1.6%)	0	0
7	The Medisafe Fit Smile™ color coded indicator feature helps me understand my blood glucose results	29 (46.8%)	26 (41.9%)	6 (9.7%)	0	0
8	Using a meter with color coded indicator feature set to my personal range helps me be more in control of my diabetes	23 (37.1%)	20 (32.3%)	19 (30.6%)	0	0
9	The Medisafe Fit Smile™ is helpful for treating low blood glucose results	11 (17.7%)	47 (75.8%)	3 (4.8%)	1 (1.6%)	0
10	Medisafe Fit Smile™ helpful for treating high blood results	26 (41.9%)	23 (37.1%)	12 (19.4%)	1 (1.6%)	0
11	The use of Blue for a low blood glucose result is intuitive and easy to understand	9 (14.5%)	38 (61.3%)	14 (22.6%)	1 (1.6%)	0
12	The Medisafe Fit Smile™ color coded indicator feature helps me make correct treatment decisions	20 (32.3%)	17 (27.4%)	24 (38.7%)	1 (1.6%)	0

## Discussion

Although HbA1c testing remains an important tool for DM management, recent studies reported that HbA1c levels should not be the main determinant of treatment effectiveness; other factors should be taken into consideration as well. On the contrary, patient satisfaction and other patient-reported outcomes should also be given significant consideration [17]. To achieve long-term stable glucose control and to reduce the risk of diabetes complications, improving patient satisfaction with treatment may play an important role in raising patients' commitment to therapy, thus improving the treatment effectiveness [18]. For example, blood glucose monitoring readings are widely recognized by patients to be difficult to interpret; but there are few studies testing how well patients perceive information or how it directly influences patient's outcomes [19,20].

Recent research has given insight on how patients with T1DM understand glucose ranges and targets data. Rankin et al. investigated the experiences of individuals with T1DM in adopting blood glucose goals and discovered that using blood glucose targets helped patients to recognize abnormalities more readily [21]. Thus, a simple CRI tailored to the appropriate BG target range by the health care providers (HCPs) may aid T1DM patients in recalling their blood glucose targets and enabling them to confidently modify it over time, thereby improving their glycemic parameters. It is evident that glucometer technology has advanced over the last few years, and research has shown that these advancements improve diabetes treatment and patient satisfaction [22,23].

The present study is the first in the MENA (the Middle East and North Africa) region to assess the patient satisfaction and clinical efficacy of BGMs featuring CRI in patients with T1DM.

A key finding of the study was clinically meaningful reductions in HbA1c observed in T1DM subjects using BGMs featuring CRI for 12 weeks with a lower frequency of hypoglycemic episodes. Most importantly, significant improvements in the GMSS subdomains of openness, emotional burden, behavioral burden were detected at the end of the study yielding a higher total of 4.33 (±0.13) after using the Medisafe Fit Smile device compared to the lower score of 2.84 (±0.22) at baseline. Additionally, the patient satisfaction with the MSCF survey at the end of this study showed a high level of satisfaction among the included T1DM patients and agreed that Medisafe Fit Smile is intuitive, easy to understand, and helpful for identifying low and high blood glucose levels. Also, most of the included patients recommended using Medisafe Fit Smile meter and strongly agreed to switch to a meter with a color-coded indicator feature.

These results are in line with our previous study that utilized a color-based smartLIGHT feature in patients with type 2 diabetes (T2DM) and showed the use of a glucometer featuring CRI was associated with improvement in the HbA1c level (not significant) and the patient satisfaction (significant) measured by GMSS as well [13]. Moreover, the recently published clinical trial showed that a glucose meter with CRI integrated with a mobile diabetes management app could significantly improve glycemic control and patient satisfaction in patients with T2DM [24]. Using these highly advanced glucometers is not only associated with higher patients satisfaction but also for healthcare professionals, as evident by Katz et al., who reported that OneTouch VerioFlex (OTVF) blood glucose monitoring system associated with wireless glucometers increases both patients' and their doctors' satisfaction [17]. The British study by Grady et al. indicated that offering automated glucose range assistance through a CRI greatly increased individuals' ability to identify blood glucose values as low, normal, or high with constant results across three distinct glucose meters used (OneTouch Select® Plus, OneTouch VerioFlex, and OneTouch Verio) in both T1DM and T2DM patients [25].

One of the limitations of this study is that majority of the participants aged less than 20-years-old. Although the relatively small sample size and no comparison group, the present study provide important data regarding one of the most advanced technologies, Medisafe Fit Smile, for diabetes management, and it is the first in Saudi Arabia and the middle east to test the effectiveness of this technology in terms of the patient’s satisfaction and glycemic control. It also provides valuable insight into the remarkable positive change found among patients with T1DM following the substitution of the Medisafe Fit Smile glucometer for the previously used methods.

## Conclusions

In conclusion, many persons with T1DM have difficulty interpreting blood glucose readings in relation to standard glycemic goal ranges. The current study supports this position, indicating that individuals with T1DM may benefit from exposure to a CRI feature such as a Medisafe Fit Smile device. Also, using a glucometer featuring CRI device has been associated with a high level of satisfaction with blood glucose measures with significant improvement in the glycemic parameters. Finally, utilizing glucometers that make glucose data easier to comprehend and understand may assist patients in making better performance and adhering to their physician’s recommendations for glycemic goals.
